# Decreased Taurine and Creatine in the Thalamus May Relate to Behavioral Impairments in Ethanol-Fed Mice: A Pilot Study of Proton Magnetic Resonance Spectroscopy

**DOI:** 10.1177/1536012117749051

**Published:** 2018-01-10

**Authors:** Su Xu, Wenjun Zhu, Yamin Wan, JiaBei Wang, Xi Chen, Liya Pi, Mary Kay Lobo, Bin Ren, Zhekang Ying, Michael Morris, Qi Cao

**Affiliations:** 1 Department of Diagnostic Radiology and Nuclear Medicine, University of Maryland School of Medicine, Baltimore, MD, USA; 2 The First Affiliated Hospital of Zhengzhou University, Zhengzhou, Henan, China; 3 Department of Pharmaceutical Sciences, University of Maryland School of Pharmacy, Baltimore, MD, USA; 4 McLean Hospital, Harvard Medical School, Belmont, MA, USA; 5 The Department of Pediatrics, University of Florida, Gainesville, FL, USA; 6 Department of Anatomy & Neurobiology, University of Maryland School of Medicine, Baltimore, MD, USA; 7 Blood Research Institute, Blood Center of Wisconsin, Department of Medicine, Medical College of Wisconsin Milwaukee, WI, USA; 8 The Department of Medicine, University of Maryland School of Medicine, Baltimore, MD, USA

**Keywords:** magnetic resonance spectroscopy, alcohol-induced minimal hepatic encephalopathy, alcoholic liver fibrosis, thalamus, taurine, creatine

## Abstract

Minimal hepatic encephalopathy (MHE) is highly prevalent, observed in up to 80% of patients with liver dysfunction. Minimal hepatic encephalopathy is defined as hepatic encephalopathy with cognitive deficits and no grossly evident neurologic abnormalities. Clinical management may be delayed due to the lack of in vivo quantitative methods needed to reveal changes in brain neurobiochemical biomarkers. To gain insight into the development of alcoholic liver disease–induced neurological dysfunction (NDF), a mouse model of late-stage alcoholic liver fibrosis (LALF) was used to investigate changes in neurochemical levels in the thalamus and hippocampus that relate to behavioral changes. Proton magnetic resonance spectroscopy of the brain and behavioral testing were performed to determine neurochemical alterations and their relationships to behavioral changes in LALF. Glutamine levels were higher in both the thalamus and hippocampus of alcohol-treated mice than in controls. Thalamic levels of taurine and creatine were significantly diminished and strongly correlated with alcohol-induced behavioral changes. Chronic long-term alcohol consumption gives rise to advanced liver fibrosis, neurochemical changes in the nuclei, and behavioral changes which may be linked to NDF. Magnetic resonance spectroscopy represents a sensitive and noninvasive measurement of pathological alterations in the brain, which may provide insight into the pathogenesis underlying the development of MHE.

## Introduction

Long-term heavy alcohol consumption is harmful to the liver and brain. Chronic alcohol consumption has been associated with a variety of cognitive impairments, ranging from central nervous system (CNS) intoxication symptoms to impaired brain functional activity, poor motor coordination, and behavioral changes.^1^ Minimal hepatic encephalopathy (MHE) is the earliest form of *hepatic encephalopathy* (HE), observed in up to 80% of patients with liver cirrhosis, thus affecting a significant population worldwide. Minimal hepatic encephalopathy has been shown to affect daily function, quality of life, driving, and overall mortality.^2^ Severe HE has the potential to be fatal and has a poor prognosis because significant damage to anatomical structures has occurred, including severe irreversible liver cirrhosis.^2^ Investigation of the mechanism of MHE and the ability to establish neurological dysfunction (NDF) caused by the effects of ethanol abuse directly on the brain or from alcoholic liver fibrosis (ALF) are limited in patients with ALF that impedes the development of clinical treatments for these conditions. In addition, the lack of reliable biomarkers that can be visualized in vivo through noninvasive imaging techniques hinders the development of early clinical diagnosis, progression monitoring, and treatment response assessment of NDF, specifically in the MHE stage. Minimal hepatic encephalopathy is the earliest stage of HE and is reversible if appropriate treatment is initiated immediately. Although significant advances in the understanding of alcohol’s effects on liver disease and neuropsychiatric disorders have been made, the neuropathogenesis of alcoholic liver disease (ALD)–related MHE is not fully understood.^3^ Emerging evidence suggests that alcohol may alter neurochemistry,^4–6^ induce brain oxidative stress,^7-8^ and alter oxidative phosphorylation in brain cells.^7^ The liver, as the key organ responsible for the breakdown of alcohol, is particularly vulnerable to the toxic byproducts of alcohol and its metabolites (eg, acetaldehyde and oxygen radicals).^9^ Another important mechanism of ALD (including ALF) is the release of the toxin lipopolysaccharides (LPS) from gut gram-negative bacteria due to chronic ethanol consumption. Lipopolysaccharides can elicit Kupffer cell activation and increase liver oxidative stress and immune over response.^10–12^ These pathological factors activate hepatic stellate cells to synthesize collagen and inhibit collagen degradation, leading to ALF formation, and subsequently to ALF-NDF. The levels of these toxins increase in the blood of patients with ALF. The toxins also can move to the brain and cause neurotoxicity.^13^ It is commonly accepted that increased exposure of the brain to ammonia released from the damaged liver, including exposure induced by late-stage alcoholic liver fibrosis (LALF), can generate an osmotic stress caused by a rise in brain glutamine (Gln).^13–15^ Until now, the specific mechanisms of the pathological progression from alcoholic MHE to end-stage HE have not been well understood. This is in part due to the lack of an appropriate animal model of ALF, along with a lack of adequate preclinical experimental studies to differentiate the roles of ALF-NDF and alcohol NDF. In addition, the lack of a noninvasive imaging modality has limited the understanding of the progress of MHE to end-stage HE. Noninvasive neuroimaging techniques in the development for monitoring these effects will contribute to the identification of early biomarkers of functional disorders before prominent tissue damage occurs, aid physicians in initiating early strategies to prevent neurotoxicity, and help monitor treatment response.

We propose the use of high resolution in vivo proton magnetic resonance spectroscopy (MRS) as a noninvasive method for the identification, visualization, and quantification of specific brain biochemical markers that reflect the underlying molecular processes of non-ALD-NDF, including MHE.^16,17^ Severe HE may present with anatomic structural damage of the brain which can be diagnosed by computed tomography (CT) and magnetic resonance imaging (MRI). However, MHE may not present with changes in the brain that can be recognized on CT and MRI. This is because patients with MHE may only have an imbalance of neurobiochemical substances within the brain and neurofunctional alteration without significant anatomical structural damage to the brain. Magnetic resonance spectroscopy enables the direct, noninvasive, in vivo assessment of the neurochemical levels of discrete brain structures and has the potential to identify the etiologies of selected brain pathologies.^18^ Several reports using MRS have shown that ethanol consumption alters brain biochemical levels in humans^19^ and animals.^20–23^ Our current animal model was established by extending the ethanol feeding length from the traditional 1 month to 7 months. This model characterizes LALF pathological damage, including significant liver fibrosis and liver dysfunction, and demonstrates behavioral disorders in MHE. Thus, this model is an appropriate model to investigate the CNS effects of alcohol and LALF. The biomarkers to be obtained by MRS will assist clinicians in diagnosing, treating, and evaluating treatment response of LALF-MHE in a huge population of patients worldwide.

To obtain a better understanding of the pathogenesis of alcohol- and LALF-related NDF that may mimic MHE, we applied in vivo localized proton MRS of the brain to a preclinical LALF model in which advanced ALF was induced following a 7-month alcohol feeding in mice. Further behavioral studies in the tested animals were carried out, and correlations were made between changes in neurochemistry and behavior resulting from this long-term alcohol feeding.

## Materials and Methods

### Animal Feeding

The model of LALF was established based on our previous methods.^9,10,24^ Female BALB/c mice were purchased from Charles River Laboratories (Wilmington, Massachusetts). At 6 to 8 weeks of age, 5 mice were housed in 1 cage in pathogen-free conditions. Mice were fed nutritionally adequate Lieber-DeCarli liquid diets (BioServ, Flemington, New Jersey) for 7 months containing 27% of calories from alcohol as the disease model. Equal calories of liquid diets with replacement of isocaloric dextrose were provided to controls. Seven animals in each group were included in our current experiments and 1 animal was not included due to inconsistency of biochemical ethanol peak profiles compared to the rest of the ethanol-fed animals. All animals were allowed free access to ethanol (study group) or control (control group) liquid diets prior to the imaging and other studies. All experimental animals were allowed free access to water and diets before MRS acquisition and the open field test (OFT). Magnetic resonance spectroscopy acquisition was performed at 7 months on all animals, both those fed ethanol and control liquid diets. Behavioral testing by OFT was performed 1 week after MRS acquisition. On the day following OFT testing, animals were sacrificed by decapitation. Blood samples and liver tissues were harvested for biochemical analysis of liver injury and histopathology, respectively. All animal procedures were approved by the Institutional Animal Care and Use Committee of University of Maryland School of Medicine.

### Pathology Assessments and Biochemistry Tests

Formalin-fixed liver sections were embedded in paraffin and sectioned (4 µm). Sections were stained with hematoxylin and eosin to examine general morphology^25,26^ and with Masson Trichrome for fibrosis.^27^ Each section was evaluated by pathologists through blinded assessment according to the Kleiner scoring system, with modifications as described in other studies.^28–30^

Plasma ethanol levels were measured by a colorimetric method using a commercial ethanol assay kit (Sigma-Aldrich, St Louis, Missouri), and plasma LPS levels were assessed by limulus amebocyte lysate test using an endotoxin assay kit (Sigma-Aldrich) as described in our previous publications.^11,31^ Plasma alanine aminotransferase (ALT) and aspartate aminotransferase (AST) assays were performed in individual mice using the photometric method, which is also described in our previous publications.^11,12^ Both AST and ALT assay kits were purchased from Sigma-Aldrich. Hyaluronic acid (HA) and α-2-macroglobulin (a2M) were measured using enzyme-linked immunosorbent assay methods^32,33^; these 2 kits also were purchased from Sigma-Aldrich.

### In Vivo Localized High-Resolution Proton MRS Experiments

In vivo MRS experiments were performed on a Bruker BioSpec 70/30USR Avance III 7 T horizontal bore MR scanner (Bruker Biospin MRI GmbH, Ettlingen, Germany) as described in our previous studies.^34,35^ A Bruker 4-element proton surface coil array was used as the receiver and a Bruker 72-mm linear volume coil as the transmitter. A 3-slice (transaxial, midsagittal, and coronal) scout image using fast low-angle shot sequence was obtained to center the mouse’s brain in the imaging field of view. A fast shimming procedure (FASTMAP) was used to improve the B_0_ homogeneity in the region of interest. Both proton density- and T_2_- weighted images were obtained for anatomic reference using a 2-dimensional rapid acquisition with relaxation enhancement sequence covering the entire brain in the coronal plane (repetition time [TR]/echo time [TE] eff_1_/TE eff_2_ = 3500/19.4/56.8 milliseconds, slice thickness = 0.5 mm, in-plane resolution = 100 × 100 µm^2^,). For proton MRS, adjustments of all first- and second-order shims over the voxel of interest were accomplished with the FASTMAP procedure. The high quality of the MR spectrum was controlled by this procedure. The shimming procedure routinely results in line widths of 6 to 9 Hz of a single ^1^H metabolites resonance (0.023-0.03 ppm). This allowed for a good separation of the glutamate (Glu; 2.35 ppm) and Gln (2.45 ppm) peaks. The triplet-like structure of taurine (Tau) has a resonance frequency primarily at 3.42 ppm. A short echo time Point-RESolved Spectroscopy pulse sequence (TR/TE = 2500/10 milliseconds, NA = 300) was used for MRS data acquisition from the hippocampus (1.5 × 6.0 × 1.5 mm^3^, number of average [NA] = 400) and the thalamus (2 × 5.5 × 1.5 mm^3^, NA = 360).^33^ The unsuppressed water signal from each of the prescribed voxels was obtained to serve as a reference for determining metabolite concentrations.

### Locomotor Activity

After completion of MRS studies, OFT was performed on all animals to examine locomotor activity.^36^ The Open field test apparatus (Med Associates Inc, St Albans, Vermont) consists of a 40 × 40 cm square box. Animals were placed in the center of the arena and allowed to explore the chamber for 60 minutes; the arena was lit throughout the test. A center zone represented the area more than 10 cm from the walls. Activity in the open field was quantified using a computer-operated activity monitoring program. Peripheral move distance (cm), peripheral ambulatory time (seconds), center entries (number of times), and center ambulatory time (seconds) were recorded. Data were divided into 10- and 20-minute intervals over the 60 minutes test session.

### Data Analysis and Statistics

Quantification of the MRS was based on frequency domain analysis using “linear combination of model spectra” (LC Model).^37^ In vivo spectra were analyzed by a superposition of a simulated basis set provided by the LC Model software (Version 6.3-0G; Stephen Provencher, Oakville, ON, Canada). The reference for determining metabolite concentration was the water signal, which was acquired from the same voxel. The metabolic profile was measured with the same parameters as biochemical data acquisition except the number of averages was set at 8. The results were normalized by the LC Model package to the metabolite concentrations and expressed as micromoles per gram wet weight (µM). Cramér-Rao lower bounds (CRLB) as reported from the LC Model analysis was used for assessing the reliability of the major metabolites. The simulated spectra of alcohol and acetate were included in the LC Model basis set. All concentrations were expressed as mean ± standard error of the mean. Only metabolites with CRLB not greater than 25% were considered in each measurement. The statistical analyses were performed on MRS and OFT results using 2-way analysis of variance with multiple comparison corrections (Bonferroni *t* test; SigmaPlot 12.5, Systat Software, Inc, San Jose, California). Pearson correlation coefficients (*r*) were calculated to identify linear dependencies between measured OFT parameters and neurochemical concentrations. Student *t* test was used for assessment of blood biochemistries. Significance level was set at *P* <.05.

## Results

### Hepatic Histopathology and Blood Biochemistry Parameters of Alcohol-Induced Liver Fibrosis

**Figure 1.**
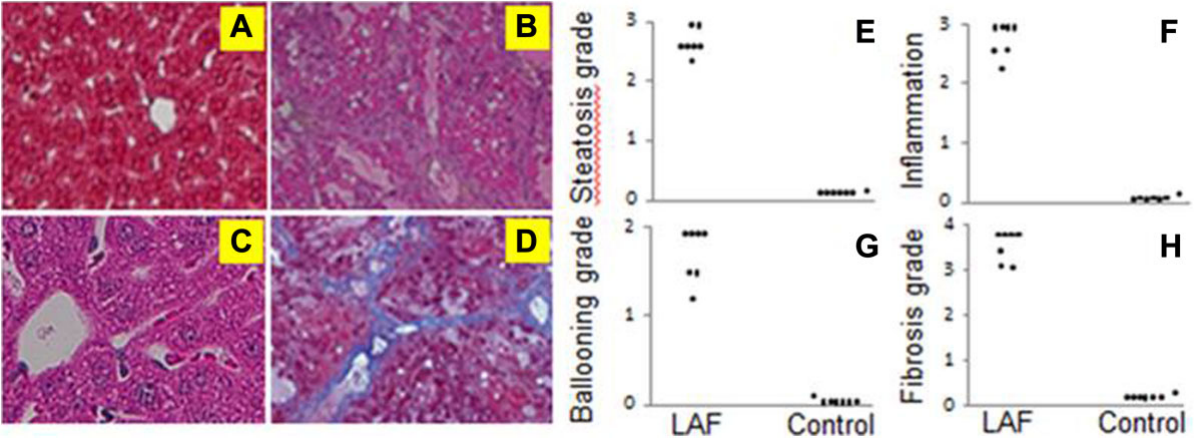
Hepatic histopathology. Hematoxylin and eosin stained liver sections in control mice (n = 7) (A) and ethanol-fed (B) mice (n = 7). Masson trichrome stain for fibrosis in control mice (C) and ethanol-fed mice (D). Magnification is 100×. There is significant increase in steatosis (E), liver inflammation (F), ballooning (G), and fibrosis (H) in ethanol-fed mice compared to controls.

Long-term alcohol consumption can induce liver pathology ranging from steatosis and inflammation to fibrosis and cirrhosis in humans.^9^ In our study, we induced alcoholic fibrosis by feeding mice an ethanol Lieber-DeCarli liquid diet for 7 months (Figure 1). Control mice received equal calories of liquid diets with isocaloric dextrose. Hematoxylin and eosin on liver sections of 7 animals in each group demonstrated no to minimal fatty liver mainly in zone 1 and inflammatory cellular infiltrates and ballooning formation in control mice (Figure 1A). Liver sections stained with Masson trichrome showed minimal fibrosis formation (Figure 1B). In contrast, ethanol-fed mice showed moderate to severe fatty liver predominantly in zone 3, significant inflammatory-cell infiltration in the pericentral area along with ballooning (Figure 1C) and severe bridging fibrosis (Figure 1D). We detected significant differences in scores of steatosis (*P* < .001, Figure 1E), inflammation (*P* < .01, Figure 1F), ballooning (*P* < .05, Figure 1G), and fibrosis (*P* < .001, Figure 1H) in the livers of the 7 mice who were fed ethanol compared to corresponding controls.

**Figure 2.**
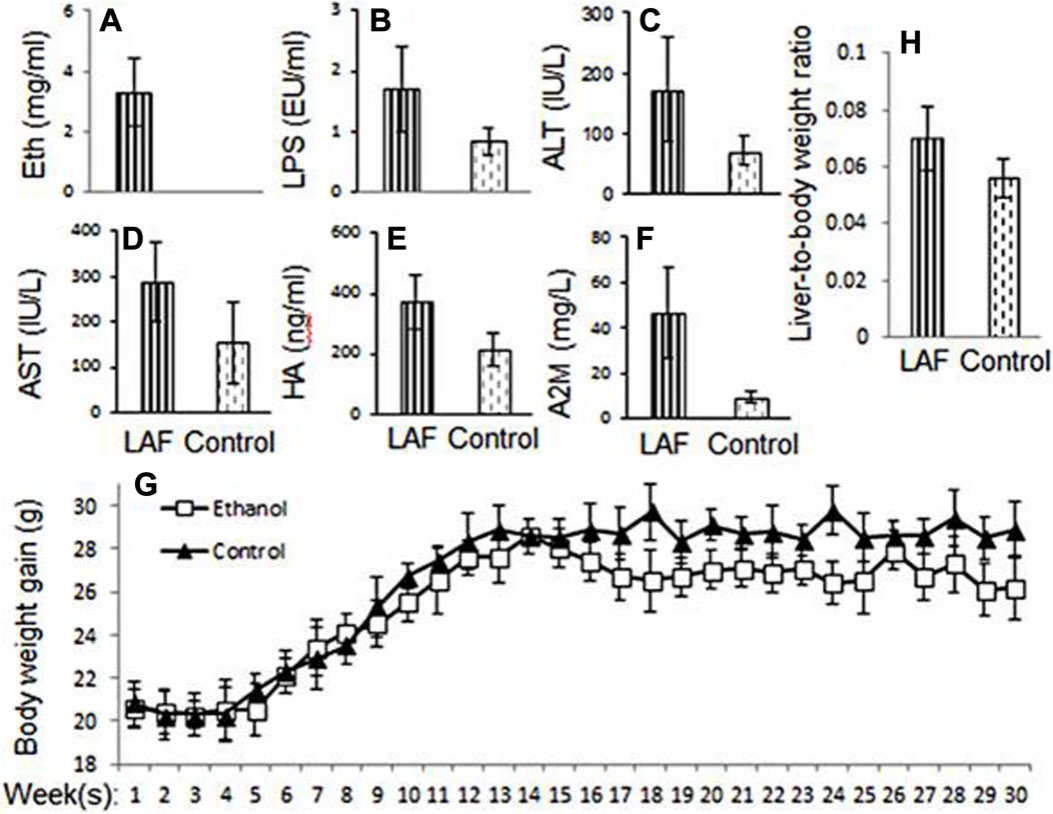
Animal model data and plasma biochemical changes. Plasma ethanol levels are persistently greater in ethanol-fed mice (A). The inflammatory mediator LPS is significantly elevated in ethanol-fed mice (B). Plasma ALT (C) and AST (D) levels are significantly increased in ethanol-fed mice compared to control mice. Serum liver fibrosis parameters HA (E) and alpha-2-macroglobulin (F) are elevated in ethanol-fed mice compared to control mice. There is a difference in liver-to-body weight ratio (H) between ethanol-fed and control animals but no significant difference in body weight gain (G). ALT indicates alanine aminotransferase; AST, aspartate aminotransferase; HA, hyaluronic acid; LPS, lipopolysaccharides.

A total of 10 mice were fed an ethanol liquid diet, and blood ethanol levels were measured at ∼3 mg/mL at the end of the experiment, whereas the corresponding 10 controls were fed isocaloric diets (Figure 2A). After 7 months, 7 mice from each group survived and were studied. Blood endotoxin LPS levels in ethanol-fed mice were greater than those of control mice (*P* <.001; Figure 2B). To assess parenchymal damage, liver enzymes ALT and AST were measured. Figure 2C and D shows an increase in plasma ALT and AST levels in mice with severe liver fibrosis compared to controls (*P* < .05). Hepatic fibrosis markers, including HA and A2M, have been reported in experimental and clinical LALF. Elevated levels of HA and A2M in ethanol-fed mice compared to controls were also observed (*P* <.01; Figure 2E and F). To assess physical changes occurring as a result of long-term ethanol feeding, weight gain and liver-to-body weight ratios were analyzed. Figure 2G and H demonstrates no significant difference in body weight gain between ethanol-fed and control mice. There was a trend toward decreased weight gain after 7 months of ethanol feeding but not before that time point. There was a significant difference in liver-to-body weight ratio between ethanol-fed and control mice (*P* <.05). These changes in blood biochemistry and ratios of liver-to-body weight further verified that the long-term uptake of ethanol resulted in significant liver pathology.

### Quality and Reproducibility of Proton MRS

Neurochemical concentrations in both the hippocampus and thalamus of the same ethanol-fed animal (n = 7) were measured twice in 1 week by proton MRS, and the results were highly reproducible. Glutamate (representing high-concentration molecules) and Gln (representing low-concentration molecules) were evaluated. Hippocampal Glu concentrations acquired from 5 ethanol-fed mice were 8.108 ± 0.101 and 8.223 ± 0.151 mM. Thalamic Glu concentrations were 9.008 ± 0.319 and 9.048 ± 0.437 mM. Hippocampal Gln concentrations were 2.843 ± 0.167 and 2.843 ± 0.201 mM. Thalamic Gln concentrations were 2.860 ± 0.097 and 2.910 ± 0.175 mM. There were no statistically significant differences between the 2 distinct time points during the same week for these 2 measurable neurochemicals, indicating that MRS quantification of neurochemicals is useful and reproducible for these experiments.

### Thalamic and Hippocampal Neurochemical Alterations in LALF Mice

**Figure 3.**
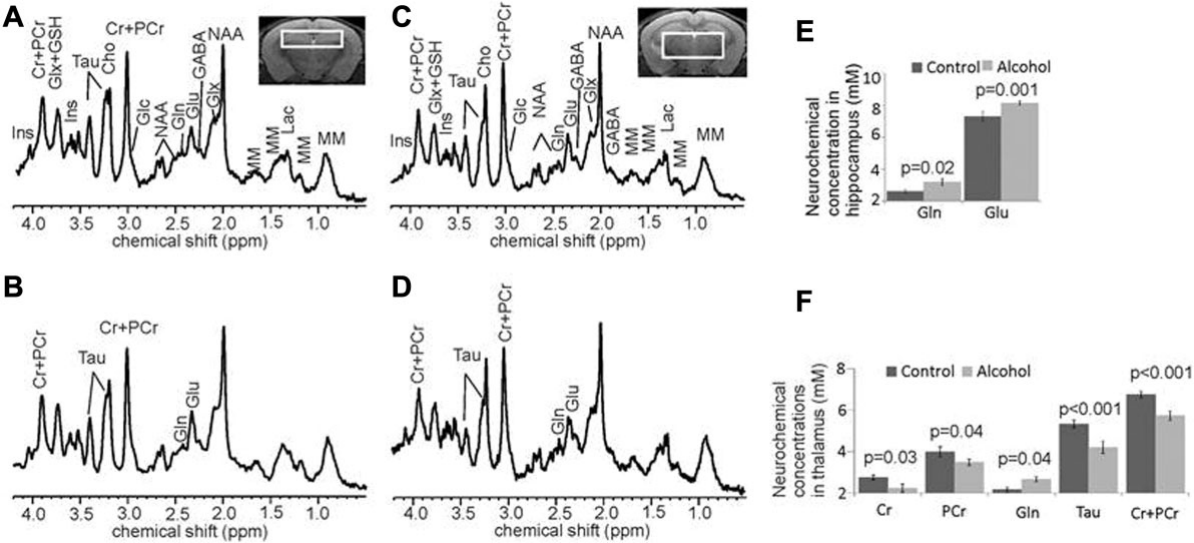
Representative in vivo ^1^H-MR spectra and corresponding voxel locations. (A) Hippocampus from an age-matched control mouse, (B) hippocampus from a 7-month-old Lieber-DeCarli alcohol liquid diet treated mouse, (C) thalamus from an age-matched control mouse, and (D) thalamus from a 7-month-old Lieber-DeCarli alcohol liquid diet–treated mouse. Comparisons of the neurochemical concentrations between control- (n = 7) and alcohol-treated (n = 7) mice in hippocampus (E) and thalamus (F). The NAA peak heights were used to normalize the MR spectra as there was no statistical difference in the NAA peak heights between the groups in the measured regions. Data presented as mean ± standard error of the mean. Ace indicates acetate; Alc, alcohol; Cho, choline-containing compounds; Cr, creatine; GABA, γ−aminobutyric acid; Glc, glucose; Gln, glutamine; Glu, glutamate; Glx, glutamate + glutamine; GSH, glutathione; Ins, *my*o-inositol; Lac, lactate; MM, macromolecules; NAA, *N*-acetylaspartate; NAAG, *N*-acetylaspartylglutamate; PCr, phosphocreatine; Tau, taurine; tCr (total creatine), Cr + PCr.

Magnetic resonance spectroscopy is a noninvasive method to assess changes in the neurochemical levels of discrete brain structures.^18^ Representative ^1^H MRS studies obtained using this method from ethanol-fed mice and age-matched control mice are shown in Figure 3. Spectra in Figure 3 were acquired from the hippocampus (A) and thalamus (C) of control mice while the spectra in Figure 3 were from the hippocampus (B) and thalamus (D) of ethanol-fed groups. In general, the alcohol signal was not detectable in all ethanol-fed and control mice. Interestingly, when 1 mouse just finished a free-feeding meal and MRS acquisition was performed immediately, in this particular mouse (data not shown), the thalamic concentration of alcohol rose to 4.45 mM; acetate was also detectable (0.36 mM). The data from this animal were excluded in our current experiments. By contrast, the rest of ethanol-fed mice did not show ethanol peaks on the MRS profile, which indicates brain imaging was not obtained in the acute stage of ingestion of ethanol diets. Compared to the control mice, the ethanol-fed mice showed higher Gln concentrations (Figure 3E and F) in both the hippocampus (3.194 ± 0.214 vs 2.607 ± 0.214 mM, *P* = .02) and the thalamus (2.678 ± 0.130 vs 2.173 ± 0.109 mM, *P* = .02). In the ethanol-fed group, levels of the excitatory neurotransmitter Glu were significantly elevated in the hippocampus (8.142 ± 0.136 vs 7.306 ± 0.310 mM, *P* = .001, Figure 3E). The thalamus of the ethanol-fed mice demonstrated significantly reduced levels of Tau (4.216 ± 0.300 vs 5.335 ± 0.187, *P* < .001), creatine (Cr; 2.250 ± 0.194 vs 2.763 ± 0.118, *P* = .04), phosphocreatine (PCr; 3.486 ± 0.148 vs 3.989 ± 0.257, *P* = .04), and total creatine (tCr; 5.736 ± 0.230 vs 6.752 ± 0.158, *P* < .001, Figure 3F). No other MRS-detectable metabolites significantly differed in this study. These results indicate that mice with ALF displayed high Gln in their hippocampi and thalami and reduced Tau, Cr, PCr, and tCr in their thalami. The CRLB were typically 2% to 4% for *N*-acetylaspartate, Glu, tCr, and total choline and 5% to 10% for Gln and Tau. These values were in the expected range compared to other previously published studies of ours^34,35^ and other groups.^38,39^

### Locomotor Functional Deficit and Its Correlation to the Neurochemical Alterations in LALF Mice

**Figure 4.**
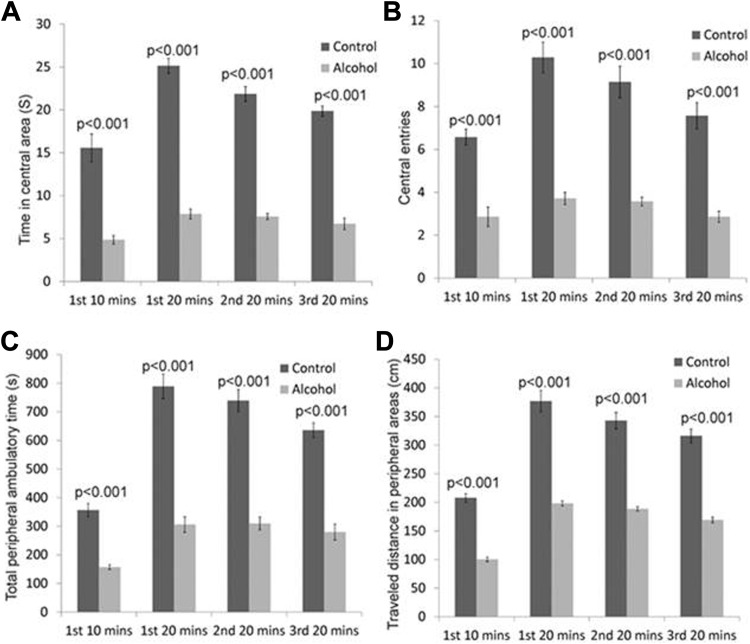
Comparison of locomotor activity by OFT between isocaloric and alcohol-treated mice (A) times in central areas, (B) numbers of central square entries, (C) total peripheral ambulatory time, and (D) traveled distance in peripheral area. Data are presented as mean ± standard error of the mean (n = 7). OFT indicates open field test.

**Figure 5.**
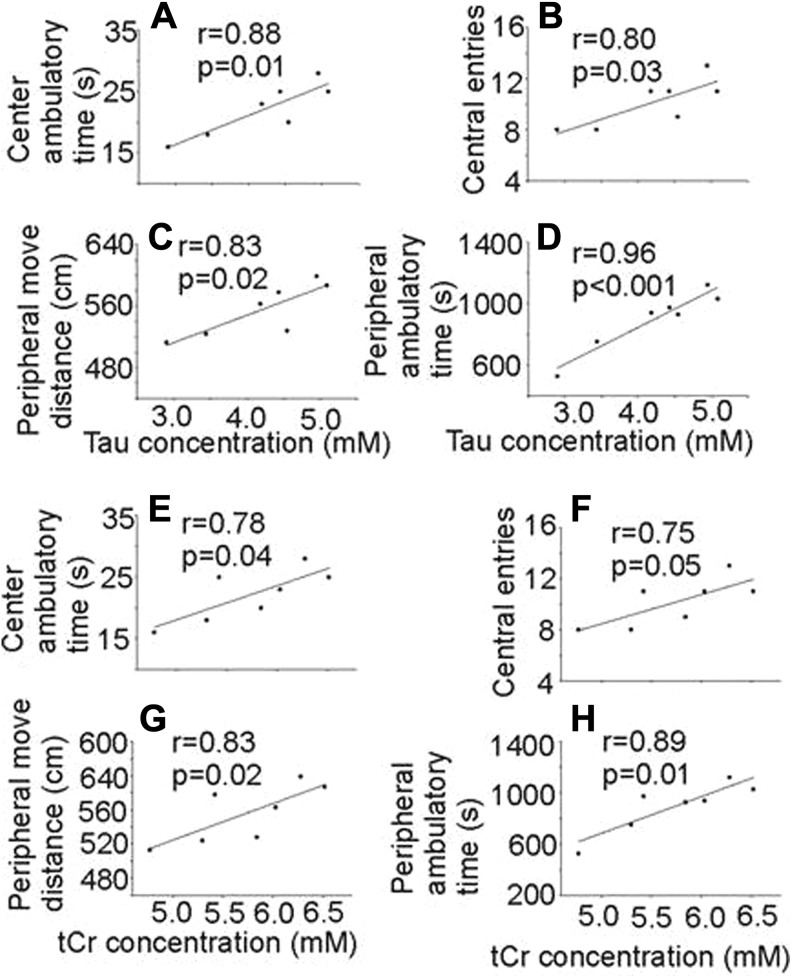
Correlation between thalamic Tau and tCr concentrations and OFT measurements. A-D, thalamic Tau and (E-H) thalamic tCr concentrations (mM). Data are presented as experiments from 7 animals. Cr indicates creatine; OFT, open field test; *r*, Pearson correlation coefficient; tCr (total creatine), Cr + PCr (phosphocreatine); Tau, taurine.

Locomotor function tests were carried out to examine behavioral changes in animals with alcoholic fibrosis. The OFT results are shown in Figure 4. Seven mice with LALF demonstrated decreased central locomotor activity compared to corresponding controls. In the total 60 minutes experimental period, there were significant differences between the 2 groups in the center ambulatory time (*F* = 544.14, *P* < .001; Figure 5A), center entries (*F* = 215.25, *P* < .001; Figure 5B), peripheral ambulatory time (*F* = 325.53, *P* ≤ .001; Figure 5C), and peripheral movement distance (*F* = 412.81, *P* < .001; Figure 5D). In addition, Figure 5 demonstrates a positive correlation between the decreased center and peripheral activities and thalamic Tau (Figure 5A-D) and tCr (Figure 5E-H) levels, respectively. Positive correlations of both Tau (*r* = 0.80-0.96, *P* = .03 to <.001) and tCr (*r* = 0.75-0.89, *P* = .05-.01) to the center and peripheral behavior changes throughout the 60-minute observation period (first 20- + second 20- + third 20-minutes) were confirmed by corresponding linear fits and 95% confidence intervals. No other correlations were observed between the other 2 neurochemicals and OFT measurements. No correlations were identified in controls between these 2 neurochemicals and OFT measurements.

### Thalamus Neuronal Degeneration, Astrocytosis, Neuronal Apoptosis, and Mitochondria Morphological Changes Without Anatomical Structural Damage in LALF Mice

**Figure 6.**
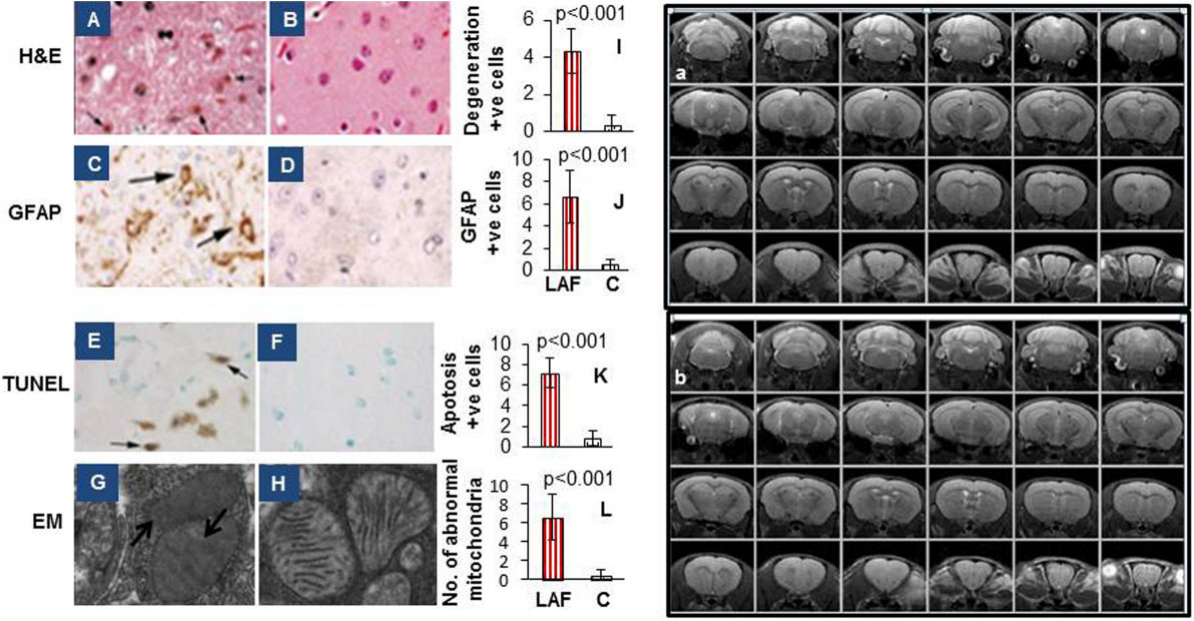
Thalamus histopathological analysis and MRI anatomic images of thalamus and brain in LALF mice. A, B, and I, H&E staining analysis of the number of positive degenerative neurons in the thalamus and quantitative data in 2 groups of LALF (A) and control (B), (C, D, and J) IHC analysis of astrocytosis in the thalamus and quantitative data in LALF (C) and control (D) animals, (E, F, and K) TUNEL staining analysis of neuron apoptosis in the thalamus, and (G, H, and L) EM analysis of mitochondrial morphological changes. The positive cell bodies (see arrows) were counted in 7 experimental animals, each group by 2 investigators in a masked manner, not knowing the classified group of sections. (H&E, 200×; IHC GFAP staining, 200×; TUNEL staining, 200×; EM, 6500× and 0.002 mm/pix). In the right panel (a) T2-weighted brain images from frontal to cerebellum of an LAF animal and (b) T2-weighted brain images from frontal to cerebellum of a control animal. There was no anatomic damage in either LALF animals or controls. EM indicates electron microscopy; GFAP, Glial fibrillary acidic protein; H&E, hematoxylin and eosin; IHC, immunohistochemistry; LALF, late-stage alcoholic liver fibrosis; MRI, magnetic resonance imaging; TUNEL, Terminal deoxynucleotidyl transferase (TdT) dUTP Nick-End Labeling.

Histological and pathological analyses were carried out to examine neuronal degeneration, astrocytosis, and neuronal apoptosis in the thalamus of animals with LALF. The histological and pathological results are shown in Figure 6. Seven mice with LALF demonstrated significant differences between the 2 groups in the neurodegeneration expressed as numbers of positive staining cells per 10 magnificent fields within the thalamus (4.1 ± 1.7 vs 0.4 ± 0.2, *P* < .001; Figure 6A, B, and I), astrocytosis (6.2 ± 2.1 vs 0.1 ± 0.2, *P* < .001; Figure 6C, D, and J), and neuron apoptosis (6.7 ± 1.2 vs 0.5 ± 0.2, *P* < .001; Figure 6E, F, and K). In addition, electron microscopy (EM) tests were performed to assess mitochondria morphological changes in animals with LALF. The EM data in Figure 6G, H, and L demonstrate significant differences between the 2 groups in mitochondria morphological changes (6.2 ± 1.2 vs 0.1 ± 0.2, *P* < .001) in the thalamus. Mitochondria with loosely packed/swollen cristae and depleted cristae are visualized in chronic ethanol-fed animals but not in controls and considered to have dysmorphological changes that may indicate that mitochondrial dysfunction is involved in the pathogenesis of LALF-NDF and ethanol-NDF. However, MRI brain images demonstrated no brain structural atrophy, and no enlargement of brain ventricles was visualized in the 2 groups (Figure 6A and B). There is no significant difference between the 2 groups in the brain weight, volume of the thalamus, and overall brain anatomic structures (data not shown), although the thalamus MRS showed biochemical changes of Tau and Cr, and the OFT had experimental evidence of depression disorder.

## Discussion

We have described neurochemical alterations in specific brain nuclei and behavioral impairments that follow long-term dietary alcohol consumption in a mouse model of LALF which may mimic MHE. The main findings for the ethanol-fed mice are as follows: (1) elevation of Gln and depletion of Tau and tCr, (2) behavioral changes correlated with decreased levels of both thalamic Tau and tCr in the thalamus of alcohol-fed animals, and (3) upregulated hippocampal glutamatergic systemic activation, as demonstrated by Glu. All of these neurochemical abnormalities quantified by MRS could contribute to the development of LALF- and ethanol-induced NDF and were associated with specific behavioral changes in the mouse model of LALF. There was no visualization of brain anatomic structural abnormalities on MRI images while in the thalamus, mitochondrial dysmorphological changes–related neuronal degeneration and apoptosis as well as astrocytosis were identified in histopathological, immunohistochemistry and EM studies.

Glutamine is the major source of nitrogen for the synthesis of nonessential amino acids, as well as nucleotides and hexosamines.^40^ Brain edema or cell swelling is one of the major neurological complications of alcohol-induced hepatic NDF. It is postulated that Gln is involved in the production of cell swelling.^41^ Glutamine is an end product of ammonia detoxification, a process that gains importance in chronic alcoholism, since ammonia toxicity is a key symptom of liver damage. Conversion of ammonia to Gln in astrocytes is a rapid, efficient process.^42,43^ Glutamine is also the breakdown product of Glu that is taken up by astrocytes following removal from the synapse. Our data suggest the increase in Glu in the hippocampus may be a contributing factor to the observed increase in Gln. However, the elevated levels of Gln can result in osmotic and oxidative stress, leading to excessive production of free radicals and induction of mitochondrial permeability (which precedes mitochondrial swelling), a phenomenon known to cause astrocyte dysfunction.^41^

Our data do not demonstrate a significant correlation between Gln and behavioral changes in either brain region. Further investigation of regional patterns of oxidative stress and mitochondrial dysfunction in the thalamus is indicated. Our results demonstrated reduced levels of Tau in the thalamus which is in agreement with an early study of chronic alcohol intake in rats.^44^ Alterations of thalamic Tau and tCr are correlated with behavioral changes in our study. Several previous studies have demonstrated a significant interaction between Tau and behavioral changes in the setting of acute alcohol ingestion in rodents.^45–48^ Acute administration of Tau has been shown to inhibit abnormal locomotor activity that was stimulated by alcohol ingestion,^45^ suggesting that Tau can alter the locomotor simulative and behavioral changes of alcohol, which agrees with our observations. It is also possible that thalamic Tau depletion results from osmotic and oxidative stresses caused either directly by the metabolites of alcohol produced in the brain and/or those derived from the damaged liver because Tau synthesis may be negatively affected in the liver in our animal model and over formation of oxidative stress in the liver may also contribute to the consumption of Tau, an important antioxidant. Taurine is known to play a osmoregulatory role in response to cellular osmotic changes.^49^ Since Tau is also an important antioxidant,^50,51^ reduced Tau levels in the thalamus might contribute to alcohol-induced osmotic and oxidative stress.

The Cr-PCr system is essential to the maintenance of energy charge, serving as a spatial and temporal energy buffer in cells with fluctuating energy demand.^52^ Chronic alcohol consumption reduces hepatic mitochondrial oxidative phosphorylation by suppressing the synthesis of key protein subunits that are encoded by mitochondrial DNA.^53,54^ Our finding of decreased tCr in the thalamus of alcohol-fed animals may be at least partially due to decreased tCr in the liver by synergistic roles of decreased synthesis of tCr and increased consumption of tCr (an important antioxidant) in the liver. A recent proton MRS study of binge alcohol exposure in rats demonstrated a decline in tCr that quickly normalized following alcohol withdrawal.^55^ Since Cr/PCr balance reflects control of the brain’s high-energy phosphate metabolism,^56^ decreased thalamic Cr and PCr levels may reflect energy depletion, which might also diminish locomotor activity.^57,58^ Future studies are planned to investigate the correlation between liver and brain Tau and tCr when interventions specifically protective against LALF are applied and to investigate effects of Tau and tCr in ALD-NDF development when supplemental Tau and tCr are applied.

The glutamatergic system also plays an important role in long-term potentiation, which is persistently increased following high-frequency stimulation of a chemical synapse and following long-term depression and anxiety. Chronic use of alcohol appears to upregulate N-methyl-d-aspartate receptor expression in the brain.^59^ Alterations in the subunit composition of Glu receptors have been observed following chronic administration of alcohol to mice.^57^ Observation of elevated hippocampal Glu in mice with LALF provides further evidence that the chronic intake of alcohol results in upregulation of the glutamatergic system, although it is not clear what role this system plays, because there is no significant correlation between Glu levels and behavioral changes. Further investigation of why Glu elevation in the thalamus and hippocampus is not associated with behavioral changes should be undertaken. One plan is to monitor progressive changes of Glu level within these 2 nuclei at different time points of alcohol feeding indicating early ALD to LALF and to seek a correlation between Glu levels and behavioral changes in a larger number of LALF animals.

Excessive ethanol consumption exerts adverse effects on many organs in the body. In the liver, hepatic Kupffer cells are activated by LPS from gram-negative bacteria due to ethanol-mediated, increased gut permeability. Furthermore, reactive oxygen species (ROS) can be generated during ethanol metabolism leading to hepatocyte injury and liver fibrosis. The effects of substantial production of these inflammatory cytokines and ROS in advanced liver fibrosis (cirrhosis) go far beyond liver damage. Over formation of inflammatory substances in the liver may explain decreased levels of liver Tau and tCr as well. These factors of inflammatory cytokines and ROS can cross the blood–brain barrier and activate brain microglial cells and endothelial cells, leading to neuroinflammation. Associations between in vivo neuroimaging and brain cytokine markers have been found in experimental models of Wernicke encephalopathy and alcohol binge exposure.^60,61^ In fact, MHE develops as a spectrum of neuropsychiatric abnormalities in many alcoholic patients with liver dysfunction during cirrhosis while brain anatomic structures may be visually normal, as seen in the current study where no abnormal brain anatomic structures were visualized on MRI. Thus, a molecular association between neurological changes and LALF following chronic ethanol intake may be postulated. Developing molecular neuroimaging techniques, such as the method of MRS described in this study for noninvasive, in vivo monitoring of these alterations will contribute to the identification of biomarkers of functional disorder before the development of significant, irreversible brain tissue damage is seen on MRI images. Identification and evaluation of the disease process through these techniques could also help to develop more effective early diagnosis strategies for MHE and help to evaluate adequate strategies to decrease neurotoxicity and delay the onset of late-stage HE, which is typically already present when there is evidence of abnormal brain anatomic structures on MRI. In this study, it was hypothesized that brain oxidative stress, altered energy metabolism, immune responses, and ammonia toxicity, may contribute to adverse brain effects caused directly by alcohol and LALF. However, the MRS findings in this pilot study of decreased Tau and Cr indicate that brain oxidative stress is one of the important pathological factors that requires further investigation. The role of brain oxidative stress effects including oxidative stress formation, regulatory signal pathway of brain oxidative stress formation, and the pathological effects of brain oxidative stress on NDF along with key pathological signal molecules are areas for future study. In addition, whether the process is reversible by stopping alcohol feeding in LALF mice for various durations deserves further investigation.

The currently described LALF model is the first reported instance where extended ethanol feeding of animals resulted in ethanol-induced LALF with features of LALF-MHE. In the literature, ALF/cirrhosis models were induced in short-term alcohol-fed mice with administration of chemicals such as carbon tetrachloride (CCl_4_),^62^ which are not feasible to investigate pure LALF-MHE because CCl_4_ obviously causes brain and other organ injuries. On the other hand, the Tsukamoto-French model has demonstrated ALF changes and liver dysfunction, but no NDF studies were reported.^63^ This gastric feeding model of ALF also requires a complicated surgical process and the feeding catheter incorporates a metal device which may limit MR imaging.

Our model shows severe liver fibrosis along with steatohepatitis similar to the findings of ALF in human alcoholics. Thus, the model may be a useful tool to further investigate mechanism of LALF-associated MHE. Another important mechanism of ALD and ALD-NDF is that LPS is released from the cell wall of gut bacteria after chronic ethanol feeding along with variations in the balance of gut bacteria colonies and increased gut wall permeability.^10–13^ Lipopolysaccharides can stimulate release of inflammatory and fibrogenic mediators such as tumor necrosis factor α, interleukin 6, and ROS by liver inflammatory cells such as Kupffer cells,^10–12^ leading to formation of ALD and ALD-NDF. At the end stage of ALD, LALF can cause MHE in the clinical setting, but the lack of an appropriate animal model limits further investigation of the pathogenesis of MHE and the investigation of potential beneficial interventions. The animal model described in this study provides a useful tool to further explore underlying mechanisms of LALF-MHE and to study correlations between other biomarkers including Gln using MRS technology and NDF by OFT and other methods in animals with a visual intact brain anatomical structure on MR imaging. Future research directions will focus on such experiments of liver–brain axis using the model. No reports of depression and anxiety disorders caused by LALF in a mouse model exist in the literature, and behavioral changes measured by OFT in our current studies may represent anxiety.^36,64,65^ Future studies will investigate depression and anxiety disorders by a forced swimming test and OFT, respectively. Regions of the thalamus and hippocampus were chosen because these nuclei were associated with MHE in clinical studies.^66–68^ However, there are no such research experiments performed in preclinical studies. The MRS technique applied has been established in our core facility to distinguish ALD-NDF and alcohol-NDF using liver protective medication that is unable to pass the blood–brain barrier in the LALF model. In addition, the roles of the cerebellum, prefrontal cortex, and striatum in LALF-related behavior changes were not completely clarified. This line of study will be continued.

## Conclusion

Our experiments demonstrate that noninvasive proton MRS provides an important means to identify and quantify neurochemical changes in specific brain regions. Taurine and Cr within the thalamus are significantly positively correlated with behavioral changes, thus Tau and Cr may potentially be used as noninvasive parameters for monitoring NDF in diagnostic and therapeutic settings. This LALF mouse model established by extended alcohol consumption may provide a promising model for future research in the field of MHE. Further investigation will examine whether neurological and/or behavioral dysfunction is caused by ethanol-associated liver fibrosis, direct ethanol injury to the CNS, or both. In addition, a deeper understanding of these processes will allow us to identify early-stage alcohol toxicity that may result from early ALD while also aiding the design of intervention strategies to ameliorate the harmful effects of alcohol on the brain and liver.

## References

[1] Brust JC Ethanol and cognition: indirect effects, neurotoxicity and neuroprotection: a review. Int J Environ Res Public Health. 2010;7(4):1540–1547. 20617045 10.3390/ijerph7041540 PMC2872345

[2] Wang FS Fan JG Zhang Z Gao B Wang HY The global burden of liver disease: the major impact of China. Hepatology. 2014;60(6):2099–2108. 25164003 10.1002/hep.27406 PMC4867229

[3] Manzo-Avalos S Saavedra-Molina A Cellular and mitochondrial effects of alcohol consumption. Int J Environ Res Public Health. 2010;7(12):4281–4304. 21318009 10.3390/ijerph7124281 PMC3037055

[4] Meyerhoff DJ Blumenfeld R Truran D Effects of heavy drinking, binge drinking, and family history of alcoholism on regional brain metabolites. Alcohol Clin Exp Res. 2004;28(4):650–661. 15100618 10.1097/01.ALC.0000121805.12350.CA PMC2365749

[5] Parks MH Dawant BM Riddle WR Longitudinal brain metabolic characterization of chronic alcoholics with proton magnetic resonance spectroscopy. Alcohol Clin Exp Res. 2002;26(9):1368–1380. 12351932 10.1097/01.ALC.0000029598.07833.2D

[6] Zahr NM Mayer D Vinco S In vivo evidence for alcohol-induced neurochemical changes in rat brain without protracted withdrawal, pronounced thiamine deficiency, or severe liver damage. Neuropsychopharmacology. 2009;34(6):1427–1442. 18704091 10.1038/npp.2008.119 PMC2669706

[7] Reddy VD Padmavathi P Kavitha G Saradamma B Varadacharyulu N Alcohol-induced oxidative/nitrosative stress alters brain mitochondrial membrane properties. Mol Cell Biochem. 2013;375(1-2):39–47. 23212448 10.1007/s11010-012-1526-1

[8] Crews FT Nixon K Mechanisms of neurodegeneration and regeneration in alcoholism. Alcohol. 2009;44(2):115–127. 10.1093/alcalc/agn079 PMC2948812 18940959

[9] de la M Hall P Lieber CS DeCarli LM Models of alcoholic liver disease in rodents: a critical evaluation. Alcohol Clin Exp Res. 2001;25(5 suppl ISBRA):254S–261S. 11391080 10.1097/00000374-200105051-00041

[10] Cao Q Mak KM Lieber CS Dilinoleoylphosphatidylcholine decreases LPS-induced TNF-alpha generation in Kupffer cells of ethanol-fed rats: respective roles of MAPKs and NF-kappaB. Biochem Biophys Res Commun. 2002;294(4):849–853. 12061785 10.1016/S0006-291X(02)00586-7

[11] Batey R Cao Q Madsen G Pang G Russell A Clancy R Decreased tumor necrosis factor-alpha and interleukin-1alpha production from intrahepatic mononuclear cells in chronic ethanol consumption and upregulation by endotoxin. Alcohol Clin Exp Res. 1998;22(1):150–156. 9514300

[12] Cao Q Batey R Pang G Clancy R Ethanol-altered liver-associated T cells mediate liver injury in rats administered concanavalin a (Con A) or lipopolysaccharide (LPS). Alcohol Clin Exp Res. 1999;23(10):1660–1667. 10549999

[13] Zakhari S Overview: how is alcohol metabolized by the body? Alcohol Res Health. 2006;29(4):245–254. 17718403 PMC6527027

[14] Cooper JLA Plum F Biochemistry and physiology of brain ammonia. Physiol Rev. 1987;67(2):440–519. 2882529 10.1152/physrev.1987.67.2.440

[15] Butterworth RF Pathogenesis of hepatic encephalopathy: new insights from neuroimaging and molecular studies. J Hepatol. 2003;39(2):278–285. 12873828 10.1016/s0168-8278(03)00267-8

[16] Meng LP Chen YC Li YH Zhu JS Ye JL Viability assessment of magnetic resonance spectroscopy for the detection of minimal hepatic encephalopathy severity. Eur J Radiol. 2015;84(10):2019–2023. 26170124 10.1016/j.ejrad.2015.06.027

[17] Ahluwalia V Wade JB Moeller FG The etiology of cirrhosis is a strong determinant of brain reserve: a multimodal magnetic resonance imaging study. Liver Transpl. 2015;21(9):1123–1132. 25939692 10.1002/lt.24163 PMC4550553

[18] Ende G Proton magnetic resonance spectroscopy: relevance of glutamate and GABA to neuropsychology. Neuropsychol Rev. 2015;25(3):315–325. 26264407 10.1007/s11065-015-9295-8

[19] Hermann D Weber-Fahr W Sartorius A Translational magnetic resonance spectroscopy reveals excessive central glutamate levels during alcohol withdrawal in humans and rats. Biol Psychiatry. 2012;71(11):1015–1021. 21907974 10.1016/j.biopsych.2011.07.034

[20] Lee DW Kim SY Lee T Ex vivo detection for chronic ethanol consumption-induced neurochemical changes in rats. Brain Res. 2012;1429:134–144. 22079322 10.1016/j.brainres.2011.10.017

[21] Lee H Holburn GH Price RR Proton MR spectroscopic studies of chronic alcohol exposure on the rat brain. J Magn Reson Imaging. 2003;18(2):147–151. 12884325 10.1002/jmri.10335

[22] Besson JA Greentree SG Foster MA Rimmington JE Effects of ethanol on the NMR characteristics of rat brain. Acute administration, dependency, and long-term effects. Br J Psychiatry. 1989;155:818–821. 2620209 10.1192/bjp.155.6.818

[23] Braunová Z Kasparová S Mlynárik V Metabolic changes in rat brain after prolonged ethanol consumption measured by 1H and 31P MRS experiments. Cell Mol Neurobiol. 2000;20(6):703–715. 11100978 10.1023/A:1007002925592 PMC11537520

[24] Cao Q Mak KM Lieber CS Dilinoleoylphosphatidylcholine decreases acetaldehyde-induced TNF-alpha generation in Kupffer cells of ethanol-fed rats. Biochem Biophys Res Commun. 2002;299(3):459–464. 12445823 10.1016/s0006-291x(02)02672-4

[25] Batey R Cao Q Pang G Clancy RL Effects of CH-100, a Chinese herbal medicine, on acute concanavalin a-mediated hepatitis in control and alcohol-fed animals. Alcohol Clin Exp Res. 2000;24(6):852–858. 10888074

[26] Cao Q Batey R Pang G Russell A Clancy R IL-6, IFN-gamma and TNF-alpha production by liver-associated T cells and acute liver injury in animals administered concanavalin A. Immunol Cell Biol. 1998;76(6):542–549. 9893032 10.1046/j.1440-1711.1998.00779.x

[27] Ren C Paronetto F Mak KM Leo MA Lieber CS Cytokeratin 7 staining of hepatocytes predicts progression to more severe fibrosis in alcohol-fed baboons, J Hepatol. 2003;38(6):770–775. 12763370 10.1016/s0168-8278(03)00144-2

[28] Kleiner DE Brunt EM Van Natta M; Nonalcoholic Steatohepatitis Clinical Research Network. Design and validation of a histological scoring system for nonalcoholic fatty liver disease. Hepatology. 2005;41(6):1313–1321. 15915461 10.1002/hep.20701

[29] Lieber CS Leo MA Mak KM Model of nonalcoholic steatohepatitis. Am J Clin Nutr. 2004;79(3):502–509. 14985228 10.1093/ajcn/79.3.502

[30] Dowman JK Laurence J Hopkins LJ Development of hepatocellular carcinoma in a murine model of nonalcoholic steatohepatitis induced by use of a high-fat/fructose diet and sedentary lifestyle. Am J Pathol. 2014;184(5):1550–1561. 24650559 10.1016/j.ajpath.2014.01.034 PMC4005975

[31] Bang FB A bacterial disease of Limulus polyphemus. Bull Johns Hopkins Hosp. 1956;98(5):325. 13316302

[32] Lieber CS Weiss DG Paronetto F ; Veterans Affairs Cooperative Study 391 Group. Value of fibrosis markers for staging liver fibrosis in patients with precirrhotic alcoholic liver disease. Alcohol Clin Exp Res. 2008;32(6):1031–1039. 18422837 10.1111/j.1530-0277.2008.00664.x

[33] Calès P Oberti F Michalak S A novel panel of blood markers to assess the degree of liver fibrosis. Hepatology. 2005;42(6):1373–1381. 16317693 10.1002/hep.20935

[34] Xu S Waddell J Zhu W In vivo longitudinal proton magnetic resonance spectroscopy on neonatal hypoxic-ischemic rat brain injury: neuroprotective effects of acetyl-L-carnitine. Magn Reson Med. 2015;74(6):1530–1542. 25461739 10.1002/mrm.25537 PMC4452442

[35] Xu S Ji Y Chen X Yang Y Gullapalli RP Masri R In vivo high-resolution localized ^1^H MR spectroscopy in the awake rat brain at 7 T. Magn Reson Med. 2012;69(4):937–943. 22570299 10.1002/mrm.24321 PMC3427395

[36] Wang J Cui Y Feng W Involvement of the central monoaminergic system in the antidepressant-like effect of catalpol in mice. Biosci Trends. 2014;8(5):248–252. 25382440 10.5582/bst.2014.01029

[37] Provencher SW Estimation of metabolite concentrations from localized in vivo proton NMR spectra. Magn Reson Med. 1993;30(6):672–679. 8139448 10.1002/mrm.1910300604

[38] Opstad KS Provencher SW Bell BA Griffiths JR Howe FA Detection of elevated glutathione in meningiomas by quantitative in vivo 1H MRS. Magn Reson Med. 2003;49(4):632–637. 12652533 10.1002/mrm.10416

[39] Nilsen LH Melø TM Saether O Witter MP Sonnewald U Altered neurochemical profile in the McGill-R-Thy1-APP rat model of Alzheimer’s disease: a longitudinal in vivo 1 H MRS study. J Neurochem. 2012;123(4):532–541. 22943908 10.1111/jnc.12003

[40] Hensley CT Wasti AT DeBerardinis RJ Glutamine and cancer: cell biology, physiology, and clinical opportunities. J Clin Invest. 2013;123(9):3678–3684. 23999442 10.1172/JCI69600 PMC3754270

[41] Albrecht J Norenberg MD Glutamine: a Trojan horse in ammonia neurotoxicity. Hepatology. 2006;44(4):788–794. 17006913 10.1002/hep.21357

[42] Sonnewald U Westergaard N Jones P Taylor A Bachelard HS Schousboe A Metabolism of [U-^13^C_5_] glutamine in cultured astrocytes studied by NMR spectroscopy: first evidence of astrocytic pyruvate recycling. J Neurochem. 1996;67(6):2566–2572. 8931491 10.1046/j.1471-4159.1996.67062566.x

[43] Yudkoff M Nissim I Pleasure D Astrocyte metabolism of [^15^N] glutamine: implications for the glutamine-glutamate cycle. J Neurochem. 1988;51(3):843–850. 2900878 10.1111/j.1471-4159.1988.tb01820.x

[44] Iwata H Matsuda T Lee E Yamagami S Baba A Effect of ethanol on taurine concentration in the brain. Experientia. 1980;36(3):332–333. 7189471 10.1007/BF01952308

[45] Aragon CMG Trudeau LE Amit Z Effect of taurine on ethanol-induced changes in open-field locomotor activity. Psychopharmacology (Berlin). 1992;107(2-3):337–340. 1615135 10.1007/BF02245158

[46] Aragon CMG Amit Z Taurine and ethanol-induced conditioned taste aversion. Pharmacol Biochem Behav. 1993;44(2):263–266. 8446659 10.1016/0091-3057(93)90460-b

[47] Iida S Hikichi M Effect of taurine on ethanol-induced sleeping-time in mice. J Stud Alcohol. 1976;37(1):19–26. 2810 10.15288/jsa.1976.37.19

[48] Ferko AP Ethanol-induced sleep time: interaction with taurine and a taurine antagonist. Pharmacol Biochem Behav. 1987;27(2):235–238. 3628438 10.1016/0091-3057(87)90564-8

[49] Thurston J Hauhart RE Dirgo JA Taurine: a role in osmotic regulation of mammalian brain and possible clinical significance. Life Sci. 1980;26(19):1561–1568. 7382728 10.1016/0024-3205(80)90358-6

[50] Ogasawara M Nakamura T Koyama Y Nemoto M Yoshida T Reactivity of taurine with aldehydes and its physiological role. Adv Exp Med Biol. 1994;359:71–78. 7887290 10.1007/978-1-4899-1471-2_8

[51] Eppler B Dawson R Jr Dietary taurine manipulations in aged male Fischer 344 rat tissue: taurine concentration, taurine biosynthesis, and oxidative markers. Biochem Pharmacol. 2001;62(1):29–39. 11377394 10.1016/s0006-2952(01)00647-5

[52] Wallimann T Tokarska-Schlattner M Schlattner U The creatine kinase system and pleiotropic effects of creatine. Amino Acid. 2001;40(5):1271–1296. 10.1007/s00726-011-0877-3 PMC3080659 21448658

[53] Cunningham CC Coleman WB Spach PI The effects of chronic ethanol consumption on hepatic mitochondrial energy metabolism. Alcohol Alcohol. 1990;25(2-3):127–136. 2142884 10.1093/oxfordjournals.alcalc.a044987

[54] Cahill A Cunningham CC Effects of chronic ethanol feeding on the protein composition of mitochondrial ribosomes. Electrophoresis. 2000;21(16):3420–3426. 11079562 10.1002/1522-2683(20001001)21:16<3420::AID-ELPS3420>3.0.CO;2-Q

[55] Zahr NM Mayer D Rohlfing T Brain injury and recovery following binge ethanol: evidence from in vivo magnetic resonance spectroscopy. Biol Psychiatry. 2010;67(9):846–854. 20044076 10.1016/j.biopsych.2009.10.028 PMC2854208

[56] Sartorius A Lugenbiel P Mahlstedt MM Ende G Schloss P Vollmayr B Proton magnetic resonance spectroscopic creatine correlates with creatine transporter protein density in rat brain. J Neurosci Methods. 2008;172(2):215–219. 18555535 10.1016/j.jneumeth.2008.04.028

[57] Holschneider DP Maarek JM Brain maps on the go: functional imaging during motor challenge in animals. Methods. 2008;45(4):255–261. 18554522 10.1016/j.ymeth.2008.04.006 PMC2561174

[58] Kalivas PW Churchill L Romanides A Involvement of the pallidal-thalamocortical circuit in adaptive behavior. Ann NY Acad Sci. 1999;877:64–70. 10415643 10.1111/j.1749-6632.1999.tb09261.x

[59] Qiang M Ticku MK Role of AP-1 in ethanol-induced N-methyl-D-aspartate receptor 2B subunit gene up-regulation in mouse cortical neurons. J Neurochem. 2005;95(5):1332–1341. 16313514 10.1111/j.1471-4159.2005.03464.x

[60] Zahr NM Alt C Mayer D Associations between in vivo neuroimaging and postmortem brain cytokine markers in a rodent model of Wernicke’s encephalopathy. Exp Neurol. 2014;261:109–119. 24973622 10.1016/j.expneurol.2014.06.015 PMC4194214

[61] Zahr NM Luong R Sullivan EV Pfefferbaum A Measurement of serum, liver, and brain cytokine induction, thiamine levels, and hepatopathology in rats exposed to a 4-day alcohol binge protocol. Alcohol Clin Exp Res. 2010;34(11):1858–1870. 20662804 10.1111/j.1530-0277.2010.01274.x PMC4393020

[62] Guo Y Liang X Meng M Hepatoprotective effects of Yulangsan flavone against carbon tetrachloride (CCl_4_-induced hepatic fibrosis in rats. Phytomedicine. 2017;33:28–35. 28887917 10.1016/j.phymed.2017.07.005

[63] Page A Paoli PP Hill SJ Alcohol directly stimulates epigenetic modifications in hepatic stellate cells. J Hepatol. 2015;62(2):388–397. 25457206 10.1016/j.jhep.2014.09.033 PMC4629846

[64] Delis F Thanos PK Rombola C Chronic mild stress increases alcohol intake in mice with low dopamine D2 receptor levels. Behavioral Neuroscience. 2013;127(1):95–105. 23148856 10.1037/a0030750

[65] Del Rosario A McDermott MM Panee J Effects of high fat diet and bamboo extract supplement on anxiety- and depression-like neurobehaviors in mice. Br J Nutr. 2012;108(7):1143–1149. 22313665 10.1017/S0007114511006738 PMC4659481

[66] Ahluwalia V Wade JB Heuman DM Enhancement of functional connectivity, working memory and inhibitory control on multi-modal brain MR imaging with rifaximin in cirrhosis: implications for the gut-liver-brain axis. Metab Brain Dis. 2014;29(4):1017–1025. 24590688 10.1007/s11011-014-9507-6 PMC4155029

[67] Spahr L Vingerhoets F Lazeyras F Magnetic resonance imaging and proton spectroscopic alterations correlate with Parkinsonian signs in patients with cirrhosis. Gastroenterology. 2000;119(3):774–781. 10982772 10.1053/gast.2000.17857

[68] Razek AA Abdalla A Ezzat A Megahed A Barakat T Minimal hepatic encephalopathy in children with liver cirrhosis: diffusion-weighted MR imaging and proton MR spectroscopy of the brain. Neuroradiology. 2014;56(10):885-891. 25060166 10.1007/s00234-014-1409-0

